# Between- and Within-Individual Sociodemographic and Psychological Determinants of PrEP Adherence Among Men Who have Sex with Men Prescribed a Daily PrEP Regimen in Wales

**DOI:** 10.1007/s10461-022-03890-4

**Published:** 2022-11-02

**Authors:** David Gillespie, Marijn de Bruin, Dyfrig A. Hughes, Richard Ma, Adam Williams, Fiona Wood, Zoë Couzens, Adam Jones, Kerenza Hood

**Affiliations:** 1 Centre for Trials Research, College of Biomedical & Life Sciences, School of Medicine, Cardiff University, Cardiff, Wales, UK; 2 Radboud University Medical Center, Institute of Health Sciences, IQ Healthcare, Nijmegen, Netherlands; 3 Centre for Health Economics and Medicines Evaluation, Bangor University, Bangor, Wales, UK; 4 Imperial College London, London, England, UK; 5 Division of Population Medicine and PRIME Centre Wales, College of Biomedical & Life Sciences, School of Medicine, Cardiff University, Cardiff, Wales, UK; 6 Public Health Wales NHS Trust, Cardiff, Wales, UK; 7 Policy, Research and International Development, Public Health Wales, Cardiff, Wales, UK

**Keywords:** HIV prevention, PrEP, Adherence, Determinants, MSM

## Abstract

We investigated the determinants of daily PrEP use and coverage of condomless anal sex (CAS) by PrEP among men who have sex with men in Wales, UK. We measured PrEP use by electronic monitors and CAS by secure online surveys. We defined PrEP use based on daily medication cap openings and coverage as CAS episodes preceded by ≥ 3 days of PrEP use and followed by ≥ 2 days of PrEP use. We included 57 participants (5463 observations). An STI diagnosis was associated with lower PrEP use but also lower PrEP coverage. Older adults had higher PrEP use. A belief that other PrEP users took PrEP as prescribed was associated with lower PrEP coverage. An STI diagnosis is an important cue for an intervention, reflecting episodes of high-risk sexual behaviour and low PrEP coverage. Other results provide a basis for the development of an evidence-informed intervention for promoting coverage of PrEP.

**Supplementary Information:**

The online version contains supplementary material available at 10.1007/s10461-022-03890-4.

## Introduction

HIV pre-exposure prophylaxis (PrEP) is a pharmacological HIV prevention option available in over 80 countries worldwide [1]. It has been demonstrated to be highly efficacious in preventing HIV acquisition in several populations, including men who have sex with men, HIV uninfected members of serodiscordant couples, and injecting drug users [2–4]. There are an increasing number of ways in which PrEP can be used, with oral tenofovir-emtricitabine being the most commonly available combination, and various regimens (e.g. daily or event-driven use) having a strong evidence-base [5, 6].

For PrEP to successfully prevent HIV-acquisition, dosing is required to cover periods where an individual may be exposed to HIV (e.g. through condomless sex or injecting drug use) [7]. As such, PrEP is not intended to be used indefinitely: people may take a break from PrEP during periods where they are unlikely to be exposed to HIV. To understand the extent to which PrEP is used as intended (i.e. adhered to), there needs to be a concurrent understanding of both medication use and potential risk exposure. Indeed, clinical trials that have not been able to demonstrate the effects of PrEP on HIV-acquisition are often linked to issues of a lack of PrEP use during high-risk sexual encounters [8–10].

The majority of previous research concerning adherence to PrEP has either used qualitative methods or focussed on medication taking alone, ignoring whether this occurred during high-risk episodes [11]. We have recently reported on levels of PrEP adherence among MSM in Wales by collecting concurrent data on PrEP use (via electronic monitors) and on condomless sexual behaviour (via brief weekly online surveys) [12]. We considered two definitions of PrEP adherence—one which focussed exclusively on medication use (i.e. percentage of doses taken over the number of days observed) and another which considered coverage of condomless anal sexual episodes (CAS) by a sufficient amount of daily PrEP, defined as CAS preceded by at least 3 days of daily PrEP and followed by at least 2 days of daily PrEP. We found rates of adherence of 66% for days with correct dosing, and 51% for CAS events adequately covered by PrEP, and observed that adherence and sexual activity varied considerably over time between- and within-individuals.

**Fig. 1:**
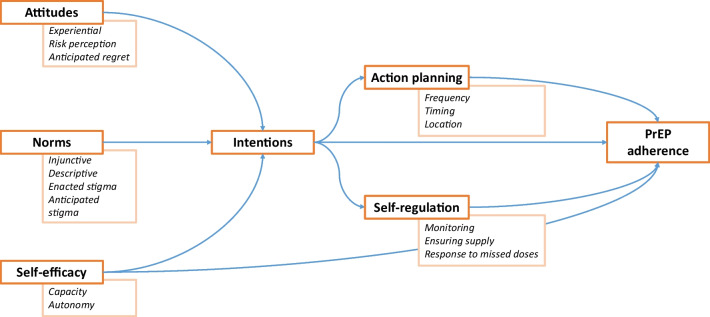
Conceptual model illustrating the studied determinants of PrEP use and PrEP coverage (collectively “PrEP adherence”) informed by the theory of planned behaviour and several proposed extensions (arrows denote theorised causal pathways)

In order to better support people to adhere to PrEP, it is important to gain insight into both behavioural patterns and the individual, social, and contextual variables associated with non-adherence to medication—especially during high-risk episodes [13, 14]. Furthermore, quantitatively examining the determinants of non-adherence to PrEP using a definition which incorporates both medication use and risk exposure enables an identification of individuals who may be most at risk of non-adherence; key drivers that may be amenable to intervention. By extending this work to study within-individual changes and their association with outcome, we will also gain better understanding of causality and the timing at which interventions may be critical. There is no established behavioural theory underpinning PrEP adherence among MSM, however, extended versions of the theory of planned behaviour have been used to explain adherence to antiretrovirals in people living with HIV, as well as other health behaviour domains [15–17]. The theory posits that attitudes, subjective norms, and perceived behavioural control predict behavioural intentions, with this intention-behaviour gap bridged by action planning and self-regulatory processes. This theory, and the quantitative items used to investigate it within this study, are illustrated in Fig. 1.

The aim of this paper is to investigate the between- and within-individual sociodemographic and psychological determinants of PrEP adherence, with adherence operationalised (i) as daily medication use and (ii) as the coverage of high-risk sexual episodes by adequate PrEP use, among individuals prescribed a daily PrEP regimen in Wales.

## Methods

### Study Design, Setting, and Participants

We conducted an ecological momentary assessment (EMA) study, using intensive longitudinal methods (i.e. using frequent repeated measurements among participants over time), of individuals accessing oral tenofovir–emtricitabine as HIV PrEP through four National Health Service (NHS) sexual health clinics in Wales, UK [12]. The NHS in Wales is a comprehensive, publicly-funded health service where PrEP has been available free-of-charge to those at-risk of HIV-acquisition since July 2017. At the time recruitment, six of the seven health boards in Wales offered PrEP through their sexual health clinics. Clinics were chosen from four of these health boards to provide a broad representation of individuals living in Wales, covering a diverse range of geographical settings and serving both urban and rural communities. All six health boards were contacted about participation—one health board declined participation and one was not enrolled due to its PrEP service being spread widely across its various clinics, with each clinic serving a small number of PrEP users.

Individuals were eligible for participation provided that they were in receipt of a prescription for oral tenofovir–emtricitabine prescription to prevent HIV-acquisition through one of the four sexual health clinics involved in the study. Individuals were excluded if they lacked capacity to consent, or were otherwise unable to carry out the study procedures (i.e. could not provide a mobile telephone number linked to a smartphone, could not use the electronic monitor supplied as part of the study, or could not provide an e-mail address). We did not exclude participants based on gender identity, sexual orientation, experience of using PrEP (new and existing PrEP users were eligible for inclusion), or PrEP regimen followed (e.g. daily or event-driven).

Participants were recruited into the EMA study between September 2019 and January 2020, with follow-up occurring between October 2019 and November 2020. For this analysis, we have restricted observations to individuals who indicated that they were following a daily PrEP regimen and to those occurring prior to 16/03/2020 (the week where control measures aiming to minimise transmission of SARS-CoV-2 were introduced in Wales). This provides a study of natural behaviour which was not impacted by the COVID-19 pandemic, and limiting observations to individuals following a daily PrEP regimen (57/60 participants indicated that they followed a daily PrEP regimen throughout the study, the other three participants followed an event-based regimen, which involved taking two pills as a single dose 2–24 h prior to condomless sexual intercourse, followed by one pill a day thereafter until two sex-free days had passed) allowed for a meaningful comparison of determinants across different outcomes.

### Procedures

**Fig. 2:**
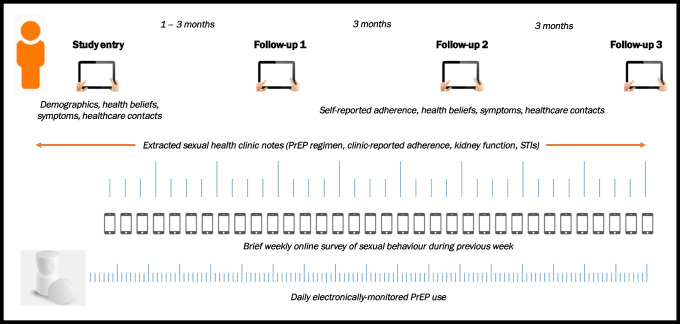
Participant flow through the study (including different data collection aspects)*. *Tick marks denote time points (daily or weekly, depending on measure)

Study procedures have been reported in full elsewhere [12]. Briefly, recruited participants were supplied with a Medication Event Monitoring System (MEMS) cap (a medication bottle cap containing an electronic monitor which records the date and time of each opening) [18, 19], and were instructed to store their PrEP medication in the original container with this cap, opening and replacing the cap only when they were taking their medication. One-week following recruitment, and weekly thereafter, participants were e-mailed a link to an online survey about condomless sex for each day in the preceding week. At recruitment and at three subsequent time points (aligned to clinic visit dates), participants were administered a questionnaire covering sociodemographics, health beliefs and behaviours, sex and relationships, potential PrEP side effects (symptoms commonly attributed to PrEP use), and healthcare contacts. Data recorded in clinic notes over the study period were also extracted. See Fig. 2 for an illustration of the participant flow through the study.

### Outcomes

We focused on two outcomes: (i) Daily PrEP use. We defined this as instances of the MEMS cap being opened at least once on an observed day (yes/no). As a summary measure, we calculated the percentage of observed days on which PrEP was taken; (ii) Coverage of CAS episodes by a daily PrEP regimen. We defined this as CAS episodes preceded by at least 3 days of daily PrEP use and followed by at least 2 days of daily PrEP use, based on pharmacokinetic and pharmacodynamics data of oral tenofovir-emtricitabine reported by Fonsart et al. [20] Thus, our summary measure was the percentage of all CAS episodes which were covered by an adequate amount of daily PrEP. We focused on CAS as our recruited sample of daily PrEP users were all men who had sex exclusively with other men (MSM).

### Candidate Determinants

We considered several potential determinants of PrEP use and PrEP coverage, grouped into three overarching themes: (i) sociodemographics (age, highest education level, presence of a chronic health condition); (ii) relationships/STI diagnoses/general HIV risk perception (relationship status, STI diagnosis, and HIV risk perception in the absence of PrEP); (iii) and psychological (components of the theory of planned behaviour [TPB] and several proposed extentions [15, 16], in addition to PrEP-related stigma [21] and HIV risk perception while taking PrEP [22]). See Supplementary Material (Table S1) for details of the original behavioural items collected as part of the study.

### Statistical Methods

The original sample size calculation was based on recruiting 60 participants, with each participant followed up for at least 7 months. Assuming some drop out and discontinuation of PrEP (i.e. an average of 160 days of PrEP use data per individual), this would have provided approximately 84% power with a two-sided alpha of 0.05 to estimate an overall average probability of PrEP use on a given day of 0.7, assuming an intra-cluster correlation coefficient of 0.6. Restricting the analysis to cover the pre-pandemic period and daily PrEP users only meant that 57 participants were included in this study, with a potential 5,928 person days covered (i.e. 104 days of follow-up per person, on average). We explored determinants of PrEP use and coverage within this sample.

Descriptive statistics are reported as frequencies and percentages, means and standard deviations (SD), and medians and interquartile ranges (IQR) as appropriate.

The model for PrEP use over time involved fitting a two-level mixed effects binary logistic regression, accounting for the correlated nature of repeated observations within individuals. An unstructured covariance matrix and robust standard errors were used to account for misspecification of model parameters. Time was modelled as time since study entry as a restricted cubic spline with three knots, specified at percentiles recommended by Harrell Jr. [23] Similarly, to model PrEP coverage over time we fitted two-level mixed effects multinomial logistic regression models with an unstructured covariance matrix and robust standard errors. Time was similarly modelled as a restricted cubic spline with three knots. The base outcome was CAS covered by PrEP, with the two other outcomes being “No CAS”, and “CAS not covered by PrEP”, thus daily observations could be characterised by one of these three states.

We examined bivariable between-individual associations with candidate determinants by fitting models which comprised an intercept, time (described above), and the candidate determinant. Most behavioural determinants were dichotomised. Stigma items were summed to provide two scores—one measuring enacted stigma (ranging from 5 to 20) and one measuring anticipated stigma (ranging from 7 to 28). Anticipated regret items were highly correlated and were combined (value = 1 if the highest regret and upset was reported on both items, 0 otherwise). Coefficients can be interpreted as the between-individual association with outcome averaged across time (i.e. whether people with higher of a particular variable also showed higher levels of PrEP use or CAS episodes not being covered by PrEP for the two models respectively). For time-varying determinants, we conducted within-individual analyses, whereby we investigated the association between prior responses and subsequent adherence outcomes by mean-centring explanatory variables and fitting between- (individual-specific mean minus grand mean) and within-individual (grand mean minus between-individual mean) variables in the models. [24]

For each outcome, the associations are reported in the following order: (i) sociodemographics; (ii) relationships/STI diagnoses/general HIV risk perception; (iii) and psychological items (grouped within the following constructs: attitudes, norms, self-efficacy, intentions, action planning, and self-regulatory processes). For the PrEP use models, we calculated pseudo R^2^ values based on McKelvey & Zavoina’s formula for each candidate determinant, with R^2^ values for psychological constructs calculated following the fitting of multivariable models within which all variables related to a specific construct were included [25].

Continuous variables (age, personalised stigma, concerns around sharing information about PrEP use with others) were modelled as linear variables following examination of model fit statistics under alternative parameterisations (polynomials and restricted cubic splines) and categorical variables were dichotomised to improve interpretation and facilitate model convergence.

Findings from the binary logistic regression models are reported as odds ratios, 95% confidence intervals, and p-values. Findings from the multinomial logistic regression models are reported as relative risk ratios (i.e. risk ratios relative to the base outcome of CAS covered by PrEP), 95% confidence intervals, and p-values. Models are based on individuals with available data (with the mixed effects models assuming data are missing at random given observed covariates) and p-values lower than 0.05 were considered statistically significant.

Statistical analyses were conducted using Stata (v17.0). [26]

## Results

### Participant Inclusion and Data Availability

PrEP use data were available for 50/57 participants (87.7% of all included participants, with the remaining seven participants not returning their MEMS caps) over 5463 person days (92.2% of days during which all 57 participants were in the study, median = 108 days, IQR 71–152 days) and concurrent PrEP use and sexual behaviour data were available for 49 participants (86.0%) over 4728 person days (79.8%, median = 89 days, IQR 61–138 days).

### Participant Characteristics

All recruited participants were cisgender and male. The majority identified as White British (50/57, 87.7%) and the median age was 35 years (IQR 28–45 years). The majority of participants were educated to degree-level or above (27/57, 47.4%). Participants had been taking PrEP for a median of 11 months at study entry (IQR 3–18 months), accessing it through sexual health clinics and via online purchase, and six were new PrEP users (i.e. prescribed PrEP for the first time during that clinic visit) at the time of recruitment (10.5%). Participants predominantly described themselves as single (44/57, 77.2%), gay (53/57, 93.0%), and having sex exclusively with other men (56/57, 98.2%) at study entry.

### Daily PrEP Use

Participants took PrEP on 67.6% of observed days (3695/5463 days), with this percentage ranging from 0% (0/119 observed days) to 100% (55/55 observed days and 52/52 observed days) between participants. The probability of taking PrEP decreased over the first 90 days of the study (from 0.79 to 0.60) before remaining relative stable thereafter (Fig. S1). The pseudo R^2^ value for the model containing the intercept and time was 0.0297 (NB this value has limited intrinsic meaning but can be used as a benchmark to interpret subsequent R^2^ values).

**Fig. 3:**
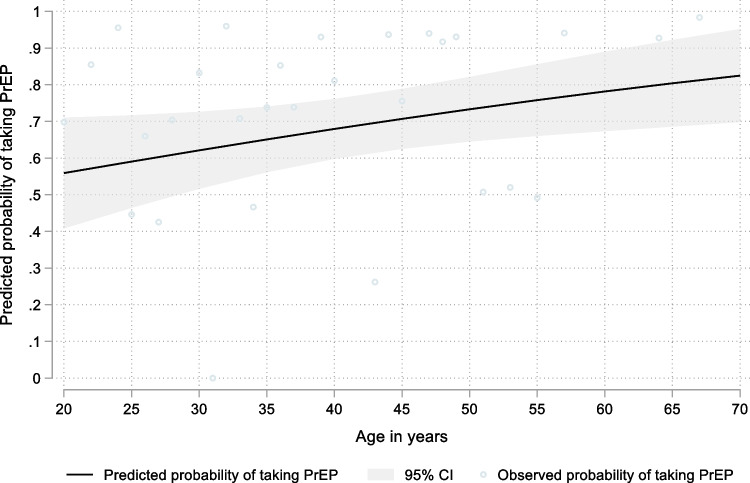
Predicted probability of daily PrEP use among MSM individuals following a daily PrEP regimen in Wales by age

Age was associated with the odds of taking PrEP on a given day, with the higher odds as age increased (OR per decade increase = 1.54, 95% CI 1.01–2.33, z = 2.03, p = 0.042). Figure 3 provides the marginal probabilities of PrEP use by age, with the predicted probability of PrEP use for a 20 year-old PrEP user being 0.56 (95% CI 0.41–0.71) and for a 50 year old PrEP user 0.73 (95% CI 0.65–0.82). There was no evidence of an association between PrEP use and the presence of a chronic health condition or education level (Table S2). Age explained the greatest proportion of the variability in PrEP use across the sociodemographic determinants (R^2^ = 0.0586).

In the between-individual analyses, participants who received an STI diagnosis, had lower odds of taking PrEP compared to those who had not (OR 0.22, 95% CI 0.13–0.39, z = − 5.26, p < 0.001). In the within-individual analysis STI diagnosis was associated with lower odds of subsequent PrEP use (OR 0.17, 95% CI 0.09–0.33, z = − 5.26, p < 0.001). A greater proportion of the variability in PrEP use was explained in the between-individual model for STI diagnosis (R^2^ = 0.0737 compared to 0.0387 in the within-individual model) (Tables S3, S5).

In the between-individual analysis, higher levels of anticipated stigma were associated with higher odds of PrEP use (OR per unit increase = 1.12, 95% CI 1.03–1.23, z = 2.60, p = 0.009). Furthermore, intentions to continue taking PrEP as prescribed were associated with higher odds of PrEP use (OR 161.02, 95% CI 8.35–3105.40, z = 3.37, p = 0.001). Intentions also explained the greatest proportion of the variability in PrEP use (R^2^ = 0.2095) compared to other between-individual constructs, with the next highest proportion of variability in PrEP use explained by behavioural norms around PrEP use (R^2^ = 0.0621) (Tables S4).

In the within-individual analysis, we found similar associations as per the between-individual analysis, with prior levels of anticipated stigma and intentions associated with subsequent PrEP use. In these models, an even greater proportion of variability in PrEP use was explained by intentions (R^2^ = 0.3086), and higher R^2^ values are noted for self-efficacy (R^2^ = 0.0945), action planning (R^2^ = 0.0735), and self-regulatory processes (R^2^ = 0.0590), compared to the between-individual analyses (Tables S6).

### Coverage of CAS Episodes by a Daily PrEP Regimen (PrEP Coverage)

Three participants reported no CAS episodes over the observation period. Overall, 53.9% of CAS episodes (207/384 CAS episodes within 46 participants) were covered by a daily PrEP regimen, with this percentage ranging from 0% for 12 participants (ranging from 1 to 34 CAS episodes per participant (median = 2 episodes, IQR 2–6 episodes) to 100% for 13 participants [ranging from 1 to 15 CAS episodes per participant (median = 4 episodes, IQR 3–6 episodes)]. The probability of CAS episodes occurring and whether they are covered by daily PrEP use, and how this varies over time, is illustrated in Fig. S2.

There was no evidence of an association between any of the sociodemographic variables and PrEP coverage (Table S7).

In the within-individual analysis, a recent STI diagnosis was associated with a higher risk of subsequent CAS episodes not being covered by PrEP (RRR relative to CAS being covered by PrEP = 3.98, 95% CI 1.05–15.09, z = 2.03, p = 0.042, Tables S8, S10).

In the between-individual analysis, high level of anticipated regret if a participant missed a dose of PrEP was associated with a higher risk of CAS episodes not being covered by PrEP (RRR relative to CAS being covered by PrEP = 4.72, 95% CI 1.21–18.39, z = 2.23, p = 0.026, Table S9).

In the within-individual analysis, participants with positive descriptive norms (i.e. responded “likely” to the statement “People who are like me take PrEP as prescribed”) had a subsequent higher risk of their CAS episodes not being covered by PrEP (RRR relative to CAS being covered by PrEP = 2.90, 95% CI 1.12–7.50, z = 2.20, p = 0.028, Table S11).

The model relating intentions to PrEP coverage would not converge. However, for 207 CAS episodes which were covered by daily PrEP, all were associated with participants indicating that they were likely to continue taking PrEP as prescribed when asked at their prior follow-up. For 175 CAS episodes which were not covered by daily PrEP, 87.4% (n = 153) were associated with participants indicating that they were likely to continue taking PrEP as prescribed when asked at their prior follow-up (with the remaining indicating that they were less than likely).

## Discussion

### Summary of Main Findings

In this EMA study of MSM prescribed HIV PrEP in Wales and following a daily regimen, we found that daily PrEP use was higher than daily PrEP coverage and found evidence of several determinants associated with both. We found that older adults had higher odds of PrEP use than younger adults, but no evidence of an association for PrEP coverage. An STI diagnosis was associated with lower PrEP use generally, but an STI diagnosis was also associated with an increased risk of subsequent CAS episodes not being covered by daily PrEP. Furthermore, we found differences and similarities across psychological determinants. Participants with intentions to continue taking PrEP as prescribed had higher odds of PrEP use (both generally, and prior intentions associated with subsequent PrEP use). Higher levels of anticipated PrEP-related stigma were associated with higher levels of PrEP use. We also found an association between higher levels of anticipated regret around missed doses of PrEP and a higher overall risk of CAS episodes not being covered by daily PrEP, and participants who believed that people like them took PrEP as prescribed at higher risk of subsequent CAS episodes not being covered by daily PrEP. In both between- and within-individual analysis, intentions explained a substantial amount of the variability in PrEP use, with pre- and post-intentional psychological constructs explaining a much lower proportion.

### Strengths and Limitations

This study included a nationally representative sample of PrEP users in Wales, who are predominantly White British MSM who have opted to follow a daily regimen [27]. Our longitudinal observational study design means we were able to measure both PrEP use and coverage of CAS episodes by a daily PrEP regimen outside of the context of a clinical trial, with the latter outcome incorporating PrEP use and potential risk exposure through condomless anal sex repeatedly over time, and had high levels of data completion for both outcomes. Furthermore, this allowed us to investigate the determinants of PrEP use and PrEP coverage between- and within-individuals over time, allowing a greater understanding of the role each determinant may have compared to a study focussing on a single point in time and being useful for informing interventions which can be adapted to changes in an individual’s attitudes, beliefs, and behaviours over time (rather than be fixed across all individuals).

While nationally representative, care must be taken when generalising these findings beyond individuals adopting a non-daily PrEP regimen (e.g. event-based dosing), or among those who are not White British MSM. The measurement of PrEP use and PrEP coverage relies on accurate use of the electronic monitors supplied as part of the study and accurate and honest recall of condomless sexual behaviour over time. Participants were shown how to use their monitors at the point of recruitment and confidentiality of responses were emphasised. However, the restrictions associated with the COVID-19 pandemic limited our ability to debrief participants at the end of the study and (for example) determine the extent to which the electronically monitored data collected reflected actual PrEP use. The pandemic also limited the length of follow-up for this study, where the focus was to investigate determinants without the influence of pandemic-related control measures (e.g. lockdowns and severe restrictions on social mixing). The uncertainty around the generalisability of determinants of PrEP use and PrEP coverage measured during a pandemic to a post-pandemic (or even post-lockdown) period led us to focus on observations collected pre-pandemic. Finally, while we measured episodes of condomless sexual behaviour, we did not record the type of partner (e.g. regular or casual partner) or any other contextual factors around the partner which may indicate the level of risk associated with the condomless sexual episode. While our data indicates that participants engaged in CAS that was not covered by daily PrEP, other risk mitigation strategies may have been employed (e.g. engaging in CAS with a regular partner whose sexual health history was known) at these times.

### Comparison with Existing Literature

A systematic review by Sidebottom et al. highlighted reasons for non-adherence reported in the literature at the time. Key reasons included risk perception, knowledge, side-effects, stigma, and decision making power [11]. Low perceived risk is an often cited barrier for PrEP uptake [28], and the sample in our study were individuals who had already been prescribed PrEP, which may in part explain this finding. PrEP-related stigma has been shown to be associated with non-adherence to PrEP [28, 29], our findings suggested that increasing levels of concern around sharing PrEP use information with others was associated with a higher probability of PrEP use. The increase in the visibility of an individual’s PrEP use may explain this finding. Our finding of higher levels of PrEP use for older PrEP users and lower levels for those diagnosed with an STI are similar to the findings from an implementation programme in New South Wales [30]. Our work expands on this by also including a definition of adherence which incorporates PrEP use and CAS episodes, finding no evidence of an association between PrEP adherence and age, but continuing to find an increase in the risk of non-adherence to PrEP for those diagnosed with an STI. Our study measured determinants informed by the theory of planned behaviour and several proposed extensions. It found strong support for behavioural intentions, with pre-intentional behaviour also contributing to PrEP adherence but limited evidence supporting post-intention behaviour. A recent study, measuring PrEP adherence via self-reported PrEP use, used the integrative model of behaviour prediction to understand the extent to which key behavioural components (many overlapping with our study) were associated with PrEP adherence, finding similar associations between perceived behavioural control (capacity) and PrEP use. [31]

### Implications

Our study highlights some important research implications, in addition to some potential implications for practice. First, our work demonstrates that PrEP use and PrEP coverage are related but distinct concepts, often with non-overlapping determinants. The determinants we found that were most strongly associated with PrEP coverage were related to risk exposure (e.g. anticipated regret, descriptive norms), with several determinants associated with PrEP use but not coverage (e.g. age, anticipated stigma). Efforts to measure, understand, and intervene on PrEP coverage should aim to consider PrEP use within the context of risk exposure, acknowledging that risk exposure is not constant over time and therefore optimising PrEP use (rather than perpetual daily PrEP use) may be a desirable goal for some [32]. Furthermore, our findings highlight subgroups of PrEP users who may require further support to ensure that their CAS episodes are covered by enough daily PrEP (e.g. those with the perception that people like them take PrEP as prescribed—which may lead to the perception that there is less need for them to take PrEP), factors that may be potentially modifiable (e.g. improving attitudes, addressing perceptions of norms, and increasing self-efficacy to improve intentions), and circumstances that may trigger the need to intervene to optimise PrEP use (e.g. at the point of an STI diagnosis). The determinants found imply an intervention which moves through the various stages outlined in Fig. 2. That is, by first targeting motivations and capabilities that drive appropriate PrEP use (i.e. within the context of risk exposure), and then focus on suitable action planning and self-regulatory processes to ensure accurate PrEP use.

## Conclusion

This study examined between- and within-individual associations between behavioural determinants and PrEP use and PrEP coverage. Several individual characteristics were identified that signal high-risk of non-adherence (be that PrEP use or PrEP coverage). These results provide a good basis for the development of interventions to promote adherence to PrEP, with an emphasis on maximising coverage of CAS episodes by PrEP and recognising the need for flexibility in response within-individuals over time.

## Supplementary Information

Below is the link to the electronic supplementary material.

Supplementary file1 (DOCX 323 kb)

## Data Availability

Data are available upon request.
