# Supplementary material for: Between- and Within-Individual Sociodemographic and Psychological Determinants of PrEP Adherence Among Men Who have Sex with Men Prescribed a Daily PrEP Regimen in Wales
Source: AIDS Behav. 2022 Nov 2;27(5):1564–72. doi: 10.1007/s10461-022-03890-4 (PMC9628468; doi:10.1007/s10461-022-03890-4)
Supplement: Supplementary file 1 — Supplementary file1 (DOCX 323 kb) [file 10461_2022_3890_MOESM1_ESM.docx]

**Between- and within-individual sociodemographic and behavioural determinants of PrEP adherence among men who have sex with men prescribed a daily PrEP regimen in Wales**

**Supplementary material**

**Contents**

**Table S1:** Psychological constructs, items, and levels used in the study

**Table S2:** Between-individual associations between sociodemographic variables and PrEP use

**Table S3:** Between-individual associations between relationship status, STI diagnoses, general HIV risk perception and PrEP use

**Table S4:** Between-individual associations between psychological measures and PrEP use

**Table S5:** Within-individual associations between relationship status, STI diagnoses, general HIV risk perception and PrEP use

**Table S6:** Within-individual associations between psychological measures and PrEP use

**Table S7:** Between-individual associations between sociodemographic variables and PrEP coverage

**Table S8:** Between-individual associations between relationship status, STI diagnoses, general HIV risk perception and PrEP coverage

**Table S9:** Between-individual associations between psychological measures and PrEP coverage

**Table S10:** Within-individual associations between relationship status, STI diagnoses, general HIV risk perception and PrEP coverage

**Table S11:** Within-individual associations between psychological measures and PrEP coverage

**Figure S1:** Predicted probability of daily PrEP use over time among MSM individuals following a daily PrEP regimen in Wales

**Figure S2:** Predicted probability of daily PrEP coverage over time among MSM individuals following a daily PrEP regimen in Wales

**Table S1:** Psychological constructs, items, and levels used in study

| **Construct** | **Item** | **Variable** | **Original levels** |
| --- | --- | --- | --- |
| Attitudes towards PrEP use | Experiential | Taking PrEP as prescribed would be | Pleasant |
|  |  |  | 2 |
|  |  |  | 3 |
|  |  |  | 4 |
|  |  |  | Unpleasant |
|  | HIV risk perception with PrEP | Thinking about the type of sex you have had in the past month, if this were to continue in a similar way for the next year, what do you think your chances would be of becoming infected with HIV if you were taking PrEP? | No chance: no possibility of becoming infected with HIV |
|  |  |  | A small chance: could happen but not likely |
|  |  |  | A moderate chance: some possibility of becoming infected with HIV |
|  |  |  | A high chance: likely to become infected with HIV |
|  | Anticipated regret | If I were to miss a dose of PrEP I would feel | Absolutely no regret |
|  |  |  | 2 |
|  |  |  | 3 |
|  |  |  | 4 |
|  |  |  | Very much regret |
|  | Anticipated affective reaction | If I were to miss a dose of PrEP I would feel | Absolutely not upset |
|  |  |  | 2 |
|  |  |  | 3 |
|  |  |  | 4 |
|  |  |  | Very much upset |
| Norms around PrEP use | Injunctive | Most people who are important to me approve of me taking PrEP as prescribed | Strongly agree |
|  |  |  | 2 |
|  |  |  | 3 |
|  |  |  | 4 |
|  |  |  | Strongly disagree |
|  |  |  | N/A - nobody knows that I take PrEP |
|  | Descriptive | Most people who are like me take PrEP as prescribed | Unlikely |
|  |  |  | 2 |
|  |  |  | 3 |
|  |  |  | 4 |
|  |  |  | Likely |
|  | Enacted stigma | People I care about stopped speaking to me after learning that I take PrEP | Strongly disagree |
|  |  |  | Disagree |
|  |  |  | Agree |
|  |  |  | Strongly agree |
|  |  | I have lost friends by telling them I take PrEP | Strongly disagree |
|  |  |  | Disagree |
|  |  |  | Agree |
|  |  |  | Strongly agree |
|  |  | I have been hurt by how people reacted to learning I take PrEP | Strongly disagree |
|  |  |  | Disagree |
|  |  |  | Agree |
|  |  |  | Strongly agree |
|  |  | I regret having told some people that I take PrEP | Strongly disagree |
|  |  |  | Disagree |
|  |  |  | Agree |
|  |  |  | Strongly agree |
|  |  | I have stopped socialising with some people due to their reactions when learning that I take PrEP | Strongly disagree |
|  |  |  | Disagree |
|  |  |  | Agree |
|  |  |  | Strongly agree |
|  | Anticipated stigma | Telling someone I take PrEP is risky | Strongly disagree |
|  |  |  | Disagree |
|  |  |  | Agree |
|  |  |  | Strongly agree |
|  |  | I work hard to keep my PrEP use a secret | Strongly disagree |
|  |  |  | Disagree |
|  |  |  | Agree |
|  |  |  | Strongly agree |
|  |  | I am very careful whom I tell that I take PrEP | Strongly disagree |
|  |  |  | Disagree |
|  |  |  | Agree |
|  |  |  | Strongly agree |
|  |  | In many areas of my life, no one knows I take PrEP | Strongly disagree |
|  |  |  | Disagree |
|  |  |  | Agree |
|  |  |  | Strongly agree |
|  |  | I worry about people discriminating against me because I take PrEP | Strongly disagree |
|  |  |  | Disagree |
|  |  |  | Agree |
|  |  |  | Strongly agree |
|  |  | I worry that people will assume that because I take PrEP I have sex with lots of people | Strongly disagree |
|  |  |  | Disagree |
|  |  |  | Agree |
|  |  |  | Strongly agree |
|  |  | I worry that people will assume that because I take PrEP I am HIV positive | Strongly disagree |
|  |  |  | Disagree |
|  |  |  | Agree |
|  |  |  | Strongly agree |
| Self-efficacy around PrEP use | Capacity | I am confident that I can take PrEP as prescribed | True |
|  |  |  | 2 |
|  |  |  | 3 |
|  |  |  | 4 |
|  |  |  | False |
|  | Autonomy | Taking PrEP as prescribed is up to me | Strongly disagree |
|  |  |  | 2 |
|  |  |  | 3 |
|  |  |  | 4 |
|  |  |  | Strongly agree |
| Intentions to continue taking PrEP as prescribed | Intentions | I intend to continue taking PrEP as prescribed | Likely |
|  |  |  | 2 |
|  |  |  | 3 |
|  |  |  | 4 |
|  |  |  | Unlikely |
| Action planning (looking at the next four weeks, I know exactly…) | Frequency | Looking at the next four-weeks, I know exactly how often I will take PrEP | Strongly disagree |
|  |  |  | 2 |
|  |  |  | 3 |
|  |  |  | 4 |
|  |  |  | Strongly agree |
|  | Timing | Looking at the next four-weeks, I know exactly at what time I will take PrEP | Strongly disagree |
|  |  |  | 2 |
|  |  |  | 3 |
|  |  |  | 4 |
|  |  |  | Strongly agree |
|  | Location | Looking at the next four-weeks, I know exactly where I will take PrEP | Strongly disagree |
|  |  |  | 2 |
|  |  |  | 3 |
|  |  |  | 4 |
|  |  |  | Strongly agree |
| Self-regulatory processes around PrEP use | Monitoring intake | I watch carefully that I take PrEP as prescribed | Never |
|  |  |  | 2 |
|  |  |  | 3 |
|  |  |  | 4 |
|  |  |  | Always |
|  | Ensuring supply continuity | I make sure that I get my new PrEP prescription at the clinic before my last prescription is finished | Never |
|  |  |  | 2 |
|  |  |  | 3 |
|  |  |  | 4 |
|  |  |  | Always |
|  | Response to missed doses | If I notice that I have not taken PrEP, I think about what the reason for that was and how I can prevent that from happening again | Never |
|  |  |  | 2 |
|  |  |  | 3 |
|  |  |  | 4 |
|  |  |  | Always |

**Table S2:** Between-individual associations between sociodemographic variables and PrEP use

| **Variable** | **Category** | **OR*** | **Lower 95% CI** | **Upper 95% CI** | **z** | **p** | **R^2†^** |
| --- | --- | --- | --- | --- | --- | --- | --- |
| Age (per decade) | | 1.54 | 1.01 | 2.33 | 2.03 | 0.042 | 0.0586 |
| Highest education level | Not educated to degree level | Reference category | | | | | 0.0383 |
|  | Educated to degree-level or above | 0.59 | 0.19 | 1.83 | -0.91 | 0.363 |  |
| Chronic health condition | No | Reference category | | | | | 0.0493 |
|  | Yes | 0.44 | 0.13 | 1.47 | -1.34 | 0.180 |  |

* Models include an intercept, time, and the corresponding item only. Estimates are odds ratios, 95% confidence intervals, z-scores, and p-values respectively. Time modelled as a restricted cubic spline with three knots (T = 11, 56, 126). Correlated observations within individuals accounted for using random effects models (4,728 observations within 49 individuals, unstructured covariance). † R^2^ values are estimated from a model containing an intercept, time, and corresponding item. McKelvey & Zavoina's pseudo R-squared value calculated. Baseline R^2^ value for model containing an intercept and time only is 0.0297.

**Table S3:** Between-individual associations between relationship status, STI diagnoses, general HIV risk perception and PrEP use

| **Variable** | **Category** | **OR*** | **Lower 95% CI** | **Upper 95% CI** | **z** | **p** | **R^2†^** |
| --- | --- | --- | --- | --- | --- | --- | --- |
| Relationship status | Not single | Reference category | | | | | 0.0341 |
|  | Single | 1.76 | 0.49 | 6.31 | 0.86 | 0.388 |  |
| STI diagnosis | No | Reference category | | | | | 0.0737 |
|  | Yes | 0.22 | 0.13 | 0.39 | -5.26 | <0.001 |  |
| HIV risk perception without PrEP | Moderate/high risk of acquiring HIV in next 12 months | Reference category | | | | | 0.0330 |
|  | No/small risk of acquiring HIV in next 12 months | 1.40 | 0.76 | 2.58 | 1.09 | 0.277 |  |

* Models include an intercept, time, and the corresponding item only. Estimates are odds ratios, 95% confidence intervals, z-scores, and p-values respectively. Time modelled as a restricted cubic spline with three knots (T = 11, 56, 126). Correlated observations within individuals accounted for using random effects models (4,728 observations within 49 individuals, unstructured covariance). † R^2^ values are estimated from a model containing an intercept, time, and corresponding item. McKelvey & Zavoina's pseudo R-squared value calculated. Baseline R^2^ value for model containing an intercept and time only is 0.0297.

**Table S4:** Between-individual associations between psychological measures and PrEP use

| **Construct** | **Item** | **Level** | **OR*** | **Lower 95% CI** | **Upper 95% CI** | **z** | **p** | **R^2†^** |
| --- | --- | --- | --- | --- | --- | --- | --- | --- |
| Attitudes towards PrEP use | Experiential | Taking PrEP as prescribed is not pleasant | Reference category | | | | | 0.0311 |
|  |  | Taking PrEP as prescribed is pleasant | 1.04 | 0.51 | 2.13 | 0.12 | 0.904 |  |
|  | HIV risk perception with PrEP | Moderate/high chance of becoming infected with HIV | Reference category | | | | |  |
|  |  | No/small chance of becoming infected with HIV | 0.85 | 0.40 | 1.77 | -0.45 | 0.656 |  |
|  | Anticipated regret | Not high level of regret if PrEP dose missed | Reference category | | | | |  |
|  |  | High level of regret if PrEP dose missed | 1.29 | 0.55 | 3.05 | 0.58 | 0.562 |  |
| Norms around PrEP use | Injunctive (most people who are important to me approve of me taking PrEP as prescribed) | Not strongly agree | Reference category | | | | | 0.0621 |
|  |  | Strongly agree | 1.32 | 0.50 | 3.51 | 0.56 | 0.572 |  |
|  | Descriptive (most people who are like me take PrEP as prescribed) | Less than likely | Reference category | | | | |  |
|  |  | Likely | 0.79 | 0.42 | 1.48 | -0.73 | 0.463 |  |
|  | Enacted stigma  (higher score = higher levels of enacted PrEP-related stigma) | | 1.15 | 1.00 | 1.33 | 1.93 | 0.053 |  |
|  | Anticipated stigma  (higher score = higher levels of anticipated PrEP-related stigma) | | 1.12 | 1.03 | 1.23 | 2.60 | 0.009 |  |
| Self-efficacy around PrEP use | Capacity (I am confident I can take PrEP as prescribed) | Not true | Reference category | | | | | 0.0357 |
|  |  | True | 2.46 | 0.43 | 14.08 | 1.01 | 0.311 |  |
|  | Autonomy (Taking PrEP as prescribed is up to me) | Not strongly agree | Reference category | | | | |  |
|  |  | Strongly agree | 1.55 | 0.83 | 2.92 | 1.37 | 0.172 |  |
| Intentions to continue taking PrEP as prescribed | Intentions | Less than likely | Reference category | | | | | 0.2095 |
|  |  | Likely | 161.02 | 8.35 | 3105.40 | 3.37 | 0.001 |  |
| Action planning (looking at the next four weeks, I know exactly…) | Frequency (how often I will take PrEP) | Not strongly agree | Reference category | | | | | 0.0443 |
|  |  | Strongly agree | 1.57 | 0.77 | 3.23 | 1.23 | 0.218 |  |
|  | Timing (what time I will take PrEP) | Not strongly agree | Reference category | | | | |  |
|  |  | Strongly agree | 1.72 | 0.83 | 3.55 | 1.47 | 0.143 |  |
|  | Location (where I will take PrEP) | Not strongly agree | Reference category | | | | |  |
|  |  | Strongly agree | 1.39 | 0.55 | 3.48 | 0.70 | 0.482 |  |
| Self-regulatory processes around PrEP use | Monitoring (I watch carefully that I take PrEP as prescribed) | Not always | Reference category | | | | | 0.0466 |
|  |  | Always | 1.48 | 0.45 | 4.85 | 0.65 | 0.514 |  |
|  | Ensuring supply continuity (I make sure that I get my new PrEP prescription at the clinic before my last prescription is finished) | Not always | Reference category | | | | |  |
|  |  | Always | 0.34 | 0.09 | 1.27 | -1.60 | 0.110 |  |
|  | Responses to missed doses (If I notice that I have not taken PrEP, I think about what the reason for that was and how I can prevent that from happening again) | Not always | Reference category | | | | |  |
|  |  | Always | 1.97 | 0.73 | 5.30 | 1.35 | 0.179 |  |

*Models include an intercept, time, and the corresponding item only. Estimates are odds ratios, 95% confidence intervals, z-scores, and p-values respectively. Time modelled as a restricted cubic spline with three knots (T = 11, 56, 126). Correlated observations within individuals accounted for using random effects models (4,728 observations within 49 individuals, unstructured covariance). †R^2^ values are estimated from a model containing an intercept, time, and all corresponding items which are related to the construct. McKelvey & Zavoina's pseudo R-squared value calculated. Baseline R^2^ value for model containing an intercept and time only is 0.0297.

**Table S5:** Within-individual associations between relationship status, STI diagnoses, general HIV risk perception and PrEP use

| **Variable** | **Category** | **OR*** | **Lower 95% CI** | **Upper 95% CI** | **z** | **p** | **R^2†^** |
| --- | --- | --- | --- | --- | --- | --- | --- |
| Relationship status | Not single | Reference category | | | | | 0.0326 |
|  | Single | 2.10 | 0.45 | 9.70 | 0.95 | 0.343 |  |
| STI diagnosis | No | Reference category | | | | | 0.0387 |
|  | Yes | 0.17 | 0.09 | 0.33 | -5.26 | <0.001 |  |
| HIV risk perception without PrEP | Moderate/high risk of acquiring HIV in next 12 months | Reference category | | | | | 0.0593 |
|  | No/small risk of acquiring HIV in next 12 months | 1.32 | 0.70 | 2.53 | 0.87 | 0.384 |  |

* Models include an intercept, time, and the corresponding item only. Estimates are odds ratios, 95% confidence intervals, z-scores, and p-values respectively. Time modelled as a restricted cubic spline with three knots (T = 11, 56, 126). Correlated observations within individuals accounted for using random effects models (4,728 observations within 49 individuals, unstructured covariance). Within-individual associations estimated by mean-centring explanatory variables and fitting between- (individual-specific mean minus grand mean) and within-individual (grand mean minus between-individual mean) variables in the model. Models are fitted to lagged time points (i.e. they are estimating the association between prior levels of these candidate determinants and subsequent levels of the outcome). † R^2^ values are estimated from a model containing an intercept, time, and corresponding item. McKelvey & Zavoina's pseudo R-squared value calculated. Baseline R^2^ value for model containing an intercept and time only is 0.0297.

**Table S6:** Within-individual associations between psychological measures and PrEP use

| **Construct** | **Item** | **Level** | **OR*** | **Lower 95% CI** | **Upper 95% CI** | **z** | **p** | **R^2†^** |
| --- | --- | --- | --- | --- | --- | --- | --- | --- |
| Attitudes towards PrEP use | Experiential | Taking PrEP as prescribed is not pleasant | Reference category | | | | | 0.0401 |
|  |  | Taking PrEP as prescribed is pleasant | 1.04 | 0.48 | 2.24 | 0.10 | 0.923 |  |
|  | HIV risk perception with PrEP | Moderate/high chance of becoming infected with HIV | Reference category | | | | |  |
|  |  | No/small chance of becoming infected with HIV | 0.82 | 0.39 | 1.76 | -0.50 | 0.617 |  |
|  | Anticipated regret | Low level of regret if PrEP dose missed | Reference category | | | | |  |
|  |  | High level of regret if PrEP dose missed | 1.53 | 0.64 | 3.67 | 0.96 | 0.336 |  |
| Norms around PrEP use | Injunctive (most people who are important to me approve of me taking PrEP as prescribed) | Not strongly agree | Reference category | | | | | 0.0464 |
|  |  | Strongly agree | 1.45 | 0.47 | 4.51 | 0.64 | 0.519 |  |
|  | Descriptive (most people who are like me take PrEP as prescribed) | Less than likely | Reference category | | | | |  |
|  |  | Likely | 0.73 | 0.37 | 1.43 | -0.91 | 0.361 |  |
|  | Enacted stigma  (higher score = higher levels of enacted PrEP-related stigma) | | 1.16 | 0.99 | 1.35 | 1.83 | 0.067 |  |
|  | Anticipated stigma  (higher score = higher levels of anticipated PrEP-related stigma) | | 1.15 | 1.02 | 1.29 | 2.38 | 0.017 |  |
| Self-efficacy around PrEP use | Capacity (I am confident I can take PrEP as prescribed) | Not true | Reference category | | | | | 0.0945 |
|  |  | True | 1.88 | 0.29 | 12.23 | 0.66 | 0.511 |  |
|  | Autonomy (Taking PrEP as prescribed is up to me) | Not strongly agree | Reference category | | | | |  |
|  |  | Strongly agree | 1.62 | 0.83 | 3.17 | 1.42 | 0.157 |  |
| Intentions to continue taking PrEP as prescribed | Intentions | Less than likely | Reference category | | | | | 0.3086 |
|  |  | Likely | 26.40 | 11.52 | 60.50 | 7.74 | <0.001 |  |
| Action planning (looking at the next four weeks, I know exactly…) | Frequency (how often I will take PrEP) | Not strongly agree | Reference category | | | | | 0.0735 |
|  |  | Strongly agree | 1.46 | 0.68 | 3.13 | 0.97 | 0.333 |  |
|  | Timing (what time I will take PrEP) | Not strongly agree | Reference category | | | | |  |
|  |  | Strongly agree | 1.65 | 0.77 | 3.52 | 1.30 | 0.195 |  |
|  | Location (where I will take PrEP) | Not strongly agree | Reference category | | | | |  |
|  |  | Strongly agree | 1.43 | 0.51 | 3.96 | 0.68 | 0.496 |  |
| Self-regulatory processes around PrEP use | Monitoring (I watch carefully that I take PrEP as prescribed) | Not always | Reference category | | | | | 0.0590 |
|  |  | Always | 1.50 | 0.41 | 5.47 | 0.62 | 0.538 |  |
|  | Ensuring supply continuity (I make sure that I get my new PrEP prescription at the clinic before my last prescription is finished) | Not always | Reference category | | | | |  |
|  |  | Always | 0.25 | 0.05 | 1.31 | -1.64 | 0.101 |  |
|  | Responses to missed doses (If I notice that I have not taken PrEP, I think about what the reason for that was and how I can prevent that from happening again) | Not always | Reference category | | | | |  |
|  |  | Always | 2.28 | 0.75 | 6.96 | 1.45 | 0.147 |  |

*Models include an intercept, time, and the corresponding item only. Estimates are odds ratios, 95% confidence intervals, z-scores, and p-values respectively. Time modelled as a restricted cubic spline with three knots (T = 11, 56, 126). Correlated observations within individuals accounted for using random effects models (4,728 observations within 49 individuals, unstructured covariance). Within-individual associations estimated by mean-centring explanatory variables and fitting between- (individual-specific mean minus grand mean) and within-individual (grand mean minus between-individual mean) variables in the model. Models are fitted to lagged time points (i.e. they are estimating the association between prior levels of these candidate determinants and subsequent levels of the outcome). †R^2^ values are estimated from a model containing an intercept, time, and all corresponding items which are related to the construct. McKelvey & Zavoina's pseudo R-squared value calculated. Baseline R^2^ value for model containing an intercept and time only is 0.0297.

**Table S7:** Between-individual associations between sociodemographic variables and PrEP coverage

| **Variable** | **Category** | **No CAS episode** | | | | **CAS episode not covered by daily PrEP** | | | |
| --- | --- | --- | --- | --- | --- | --- | --- | --- | --- |
|  |  | **RRR*** | **95% CI** | **z** | **p** | **RRR*** | **95% CI** | **z** | **p** |
| Age (per decade) | | 0.56 | 0.40 to 0.79 | -3.36 | 0.001 | 0.70 | 0.40 to 1.23 | -1.24 | 0.216 |
| Highest education level | Not educated to degree level | Reference category | | | | | | | |
|  | Educated to degree-level or above | 1.26 | 0.52 to 3.02 | 0.51 | 0.609 | 0.84 | 0.22 to 3.15 | -0.26 | 0.794 |
| Chronic health condition | No | Reference category | | | | | | | |
|  | Yes | 1.24 | 0.49 to 3.15 | 0.45 | 0.653 | 2.31 | 0.60 to 8.90 | 1.21 | 0.226 |

* Base outcome = CAS episode covered by PrEP. Time modelled as a restricted cubic spline with three knots (T = 11, 56, 126). Correlated observations within individuals accounted for using random effects models (4,728 observations within 49 individuals, unstructured covariance). Models include an intercept, time, and the corresponding item only. Estimates are relative risk ratios (i.e. risk ratios relative to the base outcome of CAS covered by PrEP), 95% confidence intervals, z-scores, and p-values respectively.

**Table S8:** Between-individual associations between relationship status, STI diagnoses, general HIV risk perception and PrEP coverage

| **Variable** | **Category** | **No CAS episode** | | | | **CAS episode not covered by daily PrEP** | | | | |
| --- | --- | --- | --- | --- | --- | --- | --- | --- | --- | --- |
|  |  | **RRR*** | **95% CI** | **z** | **p** | **RRR*** | **95% CI** | **z** | **p** |  |
| Relationship status | Not single | Reference category | | | | | | | | |
|  | Single | 1.24 | 0.48 to 3.24 | 0.45 | 0.654 | 0.51 | 0.16 to 1.63 | -1.13 | 0.259 |  |
| STI diagnosis | No | Reference category | | | | | | | | |
|  | Yes | 0.91 | 0.39 to 2.12 | -0.21 | 0.832 | 2.43 | 0.85 to 6.95 | 1.66 | 0.097 |  |
| HIV risk perception without PrEP | Moderate/high risk of acquiring HIV in next 12 months | Reference category | | | | | | | | |
|  | No/small risk of acquiring HIV in next 12 months | 1.43 | 0.68 to 2.99 | 0.94 | 0.345 | 0.65 | 0.25 to 1.68 | -0.89 | 0.371 |  |

* Base outcome = CAS episode covered by PrEP. Time modelled as a restricted cubic spline with three knots (T = 11, 56, 126). Correlated observations within individuals accounted for using random effects models (4,728 observations within 49 individuals, unstructured covariance). Models include an intercept, time, and the corresponding item only. Estimates are relative risk ratios (i.e. risk ratios relative to the base outcome of CAS covered by PrEP), 95% confidence intervals, z-scores, and p-values respectively.

**Table S9:** Between-individual associations between psychological measures and PrEP coverage

| **Construct** | **Item** | **Level** | **No CAS episode** | | | | | **CAS episode not covered by daily PrEP** | | | | | |
| --- | --- | --- | --- | --- | --- | --- | --- | --- | --- | --- | --- | --- | --- |
|  |  |  | **RRR*** | **95% CI** | **z** | **p** | **RRR*** | | **95% CI** | **z** | **p** |  |  |
| Attitudes towards PrEP use | Experiential | Taking PrEP as prescribed is not pleasant | Reference category | | | | | | | | | |  |
|  |  | Taking PrEP as prescribed is pleasant | 0.86 | 0.46 to 1.59 | -0.48 | 0.630 | 1.84 | | 0.72 to 4.68 | 1.28 | 0.200 |  |  |
|  | HIV risk perception with PrEP | Moderate/high chance of becoming infected with HIV | Reference category | | | | | | | | | |  |
|  |  | No/small chance of becoming infected with HIV | 2.87 | 1.20 to 6.87 | 2.36 | 0.018 | 2.59 | | 0.84 to 8.01 | 1.66 | 0.097 |  |  |
|  | Anticipated regret | Not high level of regret if PrEP dose missed | Reference category | | | | | | | | | |  |
|  |  | High level of regret if PrEP dose missed | 3.00 | 1.05 to 8.55 | 2.06 | 0.039 | 4.72 | | 1.21 to 18.39 | 2.23 | 0.026 |  |  |
| Norms around PrEP use | Injunctive (most people who are important to me approve of me taking PrEP as prescribed) | Not strongly agree | Reference category | | | | | | | | |  |  |
|  |  | Strongly agree | 1.53 | 0.81 to 2.88 | 1.31 | 0.192 | 1.99 | | 0.80 to 4.92 | 1.49 | 0.136 |  |  |
|  | Descriptive (most people who are like me take PrEP as prescribed) | Less than likely | Reference category | | | | | | | | |  |  |
|  |  | Likely | 1.13 | 0.68 to 1.90 | 0.48 | 0.632 | 2.09 | | 0.93 to 4.69 | 1.78 | 0.075 |  |  |
|  | Enacted stigma  (higher score = higher levels of enacted PrEP-related stigma) | | 0.99 | 0.86 to 1.14 | -0.14 | 0.888 | 0.95 | | 0.77 to 1.17 | -0.45 | 0.656 |  |  |
|  | Anticipated stigma  (higher score = higher levels of anticipated PrEP-related stigma) | | 0.98 | 0.90 to 1.06 | -0.61 | 0.542 | 0.98 | | 0.88 to 1.10 | -0.32 | 0.745 |  |  |
| Self-efficacy around PrEP use | Capacity (I am confident I can take PrEP as prescribed) | Not true | Reference category | | | | | | | | |  |  |
|  |  | True | 0.92 | 0.28 to 2.96 | -0.14 | 0.885 | 3.65 | | 0.38 to 35.28 | 1.12 | 0.263 |  |  |
|  | Autonomy (Taking PrEP as prescribed is up to me) | Not strongly agree | Reference category | | | | | | | | |  |  |
|  |  | Strongly agree | 1.52 | 0.68 to 3.39 | 1.02 | 0.306 | 1.31 | | 0.45 to 3.78 | 0.50 | 0.618 |  |  |
| Action planning (looking at the next four weeks, I know exactly…) | Frequency (how often I will take PrEP) | Not strongly agree | Reference category | | | | | | | | |  |  |
|  |  | Strongly agree | 0.83 | 0.25 to 2.79 | -0.30 | 0.761 | 0.41 | | 0.08 to 2.18 | -1.05 | 0.296 |  |  |
|  | Timing (what time I will take PrEP) | Not strongly agree | Reference category | | | | | | | | |  |  |
|  |  | Strongly agree | 0.83 | 0.38 to 1.79 | -0.48 | 0.628 | 0.52 | | 0.20 to 1.34 | -1.35 | 0.176 |  |  |
|  | Location (where I will take PrEP) | Not strongly agree | Reference category | | | | | | | | |  |  |
|  |  | Strongly agree | 0.92 | 0.50 to 1.70 | -0.25 | 0.801 | 1.62 | | 0.64 to 4.10 | 1.01 | 0.312 |  |  |
| Self-regulatory processes around PrEP use | Monitoring (I watch carefully that I take PrEP as prescribed) | Not always | Reference category | | | | | | | | |  |  |
|  |  | Always | 0.52 | 0.24 to 1.14 | -1.64 | 0.101 | 0.60 | | 0.21 to 1.72 | -0.95 | 0.340 |  |  |
|  | Ensuring supply continuity (I make sure that I get my new PrEP prescription at the clinic before my last prescription is finished) | Not always | Reference category | | | | | | | | |  |  |
|  |  | Always | 0.87 | 0.26 to 2.90 | -0.22 | 0.823 | 2.21 | | 0.52 to 9.35 | 1.08 | 0.280 |  |  |
|  | Responses to missed doses (If I notice that I have not taken PrEP, I think about what the reason for that was and how I can prevent that from happening again) | Not always | Reference category | | | | | | | | |  |  |
|  |  | Always | 0.71 | 0.36 to 1.44 | -0.94 | 0.346 | 1.43 | | 0.41 to 5.01 | 0.56 | 0.574 |  |  |

* Base outcome = CAS episode covered by PrEP. Time modelled as a restricted cubic spline with three knots (T = 11, 56, 126). Correlated observations within individuals accounted for using random effects models (4,728 observations within 49 individuals, unstructured covariance). Models include an intercept, time, and the corresponding item only. Estimates are relative risk ratios (i.e. risk ratios relative to the base outcome of CAS covered by PrEP), 95% confidence intervals, z-scores, and p-values respectively.

**Table S10:** Within-individual associations between relationship status, STI diagnoses, general HIV risk perception and PrEP coverage

| **Variable** | **Category** | **No CAS episode** | | | | **CAS episode not covered by daily PrEP** | | | |
| --- | --- | --- | --- | --- | --- | --- | --- | --- | --- |
|  |  | **RRR*** | **95% CI** | **z** | **p** | **RRR*** | **95% CI** | **z** | **p** |
| Relationship status | Not single | Reference category | | | | | | | |
|  | Single | 0.76 | 0.08 to 6.79 | -0.25 | 0.805 | 0.31 | 0.03 to 3.11 | -0.99 | 0.321 |
| STI diagnosis | No | Reference category | | | | | | | |
|  | Yes | 1.57 | 0.49 to 4.98 | 0.76 | 0.447 | 3.98 | 1.05 to 15.09 | 2.03 | 0.042 |
| HIV risk perception without PrEP | Moderate/high risk of acquiring HIV in next 12 months | Reference category | | | | | | | |
|  | No/small risk of acquiring HIV in next 12 months | 2.14 | 0.68 to 6.79 | 1.29 | 0.196 | 1.18 | 0.31 to 4.51 | 0.24 | 0.810 |

* Base outcome = CAS episode covered by PrEP. Time modelled as a restricted cubic spline with three knots (T = 11, 56, 126). Correlated observations within individuals accounted for using random effects models (4,728 observations within 49 individuals, unstructured covariance). Models include an intercept, time, and the corresponding item only. Estimates are relative risk ratios (i.e. risk ratios relative to the base outcome of CAS covered by PrEP), 95% confidence intervals, z-scores, and p-values respectively. Within-individual associations estimated by mean-centring explanatory variables and fitting between- (individual-specific mean minus grand mean) and within-individual (grand mean minus between-individual mean) variables in the model. Models are fitted to lagged time points (i.e. they are estimating the association between prior levels of these candidate determinants and subsequent levels of the outcome).

**Table S11:** Within-individual associations between psychological measures and PrEP coverage

| **Construct** | **Item** | **Level** | **No CAS episode** | | | | | **CAS episode not covered by daily PrEP** | | | | | | |
| --- | --- | --- | --- | --- | --- | --- | --- | --- | --- | --- | --- | --- | --- | --- |
|  |  |  | **RRR*** | **95% CI** | **z** | **p** | **RRR*** | | **95% CI** | **z** | **p** |  |  |  |
| Attitudes towards PrEP use | Experiential | Taking PrEP as prescribed is not pleasant | Reference category | | | | | | | | | | |  |
|  |  | Taking PrEP as prescribed is pleasant | 0.91 | 0.40 to 2.05 | -0.23 | 0.819 | 1.70 | | 0.51 to 5.68 | 0.86 | 0.390 |  |  |  |
|  | HIV risk perception with PrEP | Moderate/high chance of becoming infected with HIV | Reference category | | | | | | | | | | |  |
|  |  | No/small chance of becoming infected with HIV | 0.32 | 0.12 to 0.89 | -2.19 | 0.028 | 0.33 | | 0.10 to 1.14 | -1.75 | 0.080 |  |  |  |
|  | Anticipated regret | Not high level of regret if PrEP dose missed | Reference category | | | | | | | | | | |  |
|  |  | High level of regret if PrEP dose missed | 4.88 | 0.90 to 26.55 | 1.84 | 0.066 | 2.83 | | 0.33 to 24.52 | 0.95 | 0.344 |  |  |  |
| Norms around PrEP use | Injunctive (most people who are important to me approve of me taking PrEP as prescribed) | Not strongly agree | Reference category | | | | | | | | | |  |  |
|  |  | Strongly agree | 1.92 | 0.80 to 4.60 | 1.46 | 0.143 | 1.69 | | 0.51 to 5.56 | 0.86 | 0.389 |  |  |  |
|  | Descriptive (most people who are like me take PrEP as prescribed) | Less than likely | Reference category | | | | | | | | | |  |  |
|  |  | Likely | 1.33 | 0.73 to 2.42 | 0.93 | 0.352 | 2.90 | | 1.12 to 7.50 | 2.20 | 0.028 |  |  |  |
|  | Enacted stigma  (higher score = higher levels of enacted PrEP-related stigma) | | 0.96 | 0.80 to 1.16 | -0.38 | 0.705 | 0.93 | | 0.71 to 1.22 | -0.51 | 0.607 |  |  |  |
|  | Anticipated stigma  (higher score = higher levels of anticipated PrEP-related stigma) | | 0.93 | 0.81 to 1.07 | -0.98 | 0.329 | 0.98 | | 0.82 to 1.18 | -0.20 | 0.841 |  |  |  |
| Self-efficacy around PrEP use | Capacity (I am confident I can take PrEP as prescribed) | Not true | Reference category | | | | | | | | | |  |  |
|  |  | True | 2.62 | 0.62 to 11.11 | 1.31 | 0.190 | 3.72 | | 0.09 to 152.93 | 0.69 | 0.489 |  |  |  |
|  | Autonomy (Taking PrEP as prescribed is up to me) | Not strongly agree | Reference category | | | | | | | | | |  |  |
|  |  | Strongly agree | 2.94 | 0.85 to 10.12 | 1.71 | 0.087 | 1.56 | | 0.35 to 6.95 | 0.59 | 0.558 |  |  |  |
| Action planning (looking at the next four weeks, I know exactly…) | Frequency (how often I will take PrEP) | Not strongly agree | Reference category | | | | | | | | | |  |  |
|  |  | Strongly agree | 0.87 | 0.14 to 5.45 | -0.15 | 0.878 | 0.23 | | 0.02 to 2.42 | -1.22 | 0.221 |  |  |  |
|  | Timing (what time I will take PrEP) | Not strongly agree | Reference category | | | | | | | | | |  |  |
|  |  | Strongly agree | 1.52 | 0.48 to 4.86 | 0.71 | 0.479 | 0.85 | | 0.23 to 3.17 | -0.24 | 0.811 |  |  |  |
|  | Location (where I will take PrEP) | Not strongly agree | Reference category | | | | | | | | | |  |  |
|  |  | Strongly agree | 1.06 | 0.49 to 2.29 | 0.16 | 0.876 | 1.84 | | 0.58 to 5.78 | 1.04 | 0.299 |  |  |  |
| Self-regulatory processes around PrEP use | Monitoring (I watch carefully that I take PrEP as prescribed) | Not always | Reference category | | | | | | | | | |  |  |
|  |  | Always | 0.77 | 0.30 to 1.98 | -0.55 | 0.585 | 0.57 | | 0.17 to 1.94 | -0.89 | 0.372 |  |  |  |
|  | Ensuring supply continuity (I make sure that I get my new PrEP prescription at the clinic before my last prescription is finished) | Not always | Reference category | | | | | | | | | |  |  |
|  |  | Always | 0.38 | 0.08 to 1.83 | -1.21 | 0.226 | 1.07 | | 0.18 to 6.37 | 0.08 | 0.938 |  |  |  |
|  | Responses to missed doses (If I notice that I have not taken PrEP, I think about what the reason for that was and how I can prevent that from happening again) | Not always | Reference category | | | | | | | | | |  |  |
|  |  | Always | 0.56 | 0.24 to 1.32 | -1.32 | 0.188 | 1.36 | | 0.19 to 9.49 | 0.30 | 0.761 |  |  |  |

* Base outcome = CAS episode covered by PrEP. Time modelled as a restricted cubic spline with three knots (T = 11, 56, 126). Correlated observations within individuals accounted for using random effects models (4,728 observations within 49 individuals, unstructured covariance). Models include an intercept, time, and the corresponding item only. Estimates are relative risk ratios (i.e. risk ratios relative to the base outcome of CAS covered by PrEP), 95% confidence intervals, z-scores, and p-values respectively. Within-individual associations estimated by mean-centring explanatory variables and fitting between- (individual-specific mean minus grand mean) and within-individual (grand mean minus between-individual mean) variables in the model. Models are fitted to lagged time points (i.e. they are estimating the association between prior levels of these candidate determinants and subsequent levels of the outcome).

**Figure S1:** Predicted probability of daily PrEP use over time among MSM individuals following a daily PrEP regimen in Wales

**Figure S2:** Predicted probability of daily PrEP coverage over time among MSM individuals following a daily PrEP regimen in Wales
